# *Rhus coriaria* induces autophagic and apoptotic cell death in pancreatic cancer cells

**DOI:** 10.3389/fphar.2024.1412565

**Published:** 2024-07-30

**Authors:** Yassine El Mahi, Zohra Nausheen Nizami, Adil Farooq Wali, Aysha Al Neyadi, Mohamed Magramane, Mazoun Al Azzani, Kholoud Arafat, Samir Attoub, Ali H. Eid, Rabah Iratni

**Affiliations:** ^1^ Department of Biology, College of Science, United Arab Emirates University, Al Ain, United Arab Emirates; ^2^ Department of Pharmaceutical Chemistry, RAK College of Pharmacy, RAK Medical and Health Sciences University, Ras Al Khaimah, United Arab Emirates; ^3^ Department of Pharmacology and Therapeutics, College of Medicine and Health Sciences, United Arab Emirates University, Al Ain, United Arab Emirates; ^4^ Department of Basic Medical Sciences, College of Medicine, QU Health, Qatar University, Doha, Qatar

**Keywords:** *Rhus coriaria*, pancreatic cancer, autophagy, apoptosis, Akt/mTOR pathway

## Abstract

**Background:**Pancreatic cancer is a leading cause of cancer-related mortality worldwide with increasing global incidence. We previously reported the anticancer effect of *Rhus coriaria* ethanolic extract (RCE) in triple negative breast and colon cancer cells. Herein, we investigated the anticancer effect of RCE on human pancreatic cancer cells.

**Methods:** Cell viability was measured using Cell Titer-Glo and staining of viable and dead cells based on differential permeability to two DNA binding dyes. Cell cycle distribution and annexin V staining was carried out in Muse cell analyzer. Protein level was determined by Western blot. Tumor growth was assessed by in ovo chick embryo chorioallantoic membrane assay.

**Results:** We found that RCE significantly inhibited the viability and colony growth of pancreatic cancer cells (Panc-1, Mia-PaCa-2, S2-013, AsPC-1). The antiproliferative effects of RCE in pancreatic cancer cells (Panc-1 and Mia-PaCa-2) were mediated through induction of G1 cell cycle arrest, Beclin-1-independent autophagy, and apoptosis. RCE activated both the extrinsic and intrinsic pathways of apoptosis and regulated the Bax/Bcl-2 apoptotic switch. Mechanistically, we found that RCE inhibited the AKT/mTOR pathway, downstream of which, inactivation of the cell cycle regulator p70S6K and downregulation of the antiapoptotic protein survivin was observed. Additionally, we found that RCE-induced autophagy preceded apoptosis. Further, we confirmed the anticancer effect of RCE in a chick embryo xenograft model and found that RCE inhibited the growth of pancreatic cancer xenografts without affecting embryo survival.

**Conclusion:** Collectively, our findings demonstrate that *Rhus coriaria* exerts potent anti-pancreatic cancer activity though cell cycle impairment, autophagy, and apoptosis, and is hence a promising source of anticancer phytochemicals.

## 1 Introduction

Pancreatic cancer is among the top ten causes of cancer-related mortality in over 130 countries (Cabasag et al., 2021; Sung et al., 2021). Concerningly, the incidence of pancreatic cancer is increasing globally, and while the death rate for other cancers, including breast, lung, and colorectal cancers is declining, there has been dismal progress on this end for pancreatic cancer (Siegel et al., 2023). This can be attributed to several factors including advanced stage of disease at diagnosis, which decreases the effectiveness of existing therapeutic approaches (Park et al., 2021; Halbrook et al., 2023).

In recent years, there has been increasing research on natural products and their derivatives as anticancer drugs (Newman and Cragg, 2020). Our lab previously reported on the anticancer effect of *Rhus coriaria* (Sumac), a Mediterranean plant whose dried fruit is used widely in Mediterranean cuisine as a spice. Various parts of *R. coriaria*, including its fruits, are used traditionally as a medicinal herb for a wide range of conditions, including ulcers, bacterial infections, and even chronic conditions, such as diabetes. In fact, various biological activities of extracts of *R. coriaria* fruits (RCE) have been reported including anti-oxidant and anti-inflammatory effects, which can be attributed to its phytochemical composition as over 200 phytochemical compounds have been characterized in *R. coriaria* extracts (Elagbar et al., 2020; Alsamri et al., 2021).

In 2015, we were the first to characterize the anticancer activity of RCE. We reported that RCE induces G1 cell cycle arrest, senescence, and Beclin-1 dependent autophagy in triple-negative breast cancer cells through activation of the p38 and ERK1/2 pathways (El Hasasna et al., 2015). Later, we reported that RCE decreases the migration and invasion capacities of triple negative breast cancer cells both *in vitro* and *in vivo* (chick embryo model). Additionally, we reported that it decreases the production of pro-inflammatory cytokines in triple-negative breast cancer cells, and inhibits angiogenesis, and that these effects are mediated through inhibition of the NFκB, STAT3, and nitric oxide pathways (El Hasasna et al., 2016). We have since also reported the anticancer activity of RCE against colon cancer cells. RCE was found to induce Beclin-1-independent autophagy and caspase 7-dependent apoptosis through proteasome-mediated degradation of Beclin-1, mTORC1, and AKT, and pro-caspase-3, respectively in colon cancer cells (Athamneh et al., 2017).

While other groups have reported on the anticancer effects of RCE against breast (Ghorbani et al., 2018; Kubatka et al., 2020; Gabr and Alghadir, 2021), prostrate (Gabr and Alghadir, 2021), and ovarian cancers (Gabr and Alghadir, 2021), the anticancer effect of RCE against pancreatic cancer has not yet been characterized. Hence, in the present study, we investigated the anticancer effect of RCE against human pancreatic cancer cells. Our results demonstrate that RCE induces its anticancer effects in human pancreatic cancer cells through induction of G1 cell cycle arrest, Beclin-1-dependent autophagy, and apoptosis. These effects are mediated through inhibition of the AKT/mTOR pathway, inactivation of p-p70S6K, and downregulation of survivin.

## 2 Materials and methods

### 2.1 Cell culture

Human pancreatic ductal adenocarcinoma cell lines, Panc-1, Mia-PaCa-2, S2-013, and AsPC-1 were cultured in DMEM (Hyclone, Cramlington, United Kingdom) supplemented with 10% heat-inactivated fetal bovine serum (Hyclone) and 100 U/mL penicillin/streptomycin (Hyclone). All cell lines were maintained in a 5% CO_2_ atmosphere at 37°C.

### 2.2 Antibodies

Primary antibodies against phosphorylated-AKT (4060S), AKT (9272S), p27 (3686S), phosphorylated-mTORC1 (2971), mTORC1 (2972), Cyclin E1 (20808), Caspase 8 (9746), Beclin-1 (3495), LC3A/B I/II (12,741), P70S6 Kinase (P70S6K; 9202S), phosphorylated-P70S6K (9205S), Bcl-2 (2872S), Bax (2772S), and Survivin (2803S) were purchased from Cell Signaling Technology (Danvers, MA, United States); against p21 (05–655), Cyclin B1 (05–373), Cyclin D1 (04–1151), and Caspase 9 (05–572) were purchased from Millipore (Hayward, CA, United States); and against p62 (Ab101266) and cleaved PARP (Ab4830) were purchased from Abcam (Cambridge, United Kingdom). Horseradish peroxidase (HRP)-conjugated goat anti-mouse (sc-2005) and goat anti-rabbit (sc-2030) antibodies, and HRP-conjugated antibodies against β-actin (sc-47778 HRP) were purchased from Santa Cruz Biotechnology (Dallas, TX, United States).

### 2.3 Preparation of ethanolic extract of *Rhus coriaria* (RCE)

Fruits of *R. coriaria* were harvested from local farm in Ajloun Mountains region, Jordan. *Rhus coriaria* extract (RCE) was prepared as described previously (El Hasasna et al., 2015). Briefly, 10 g of the dried fruits was crushed into a fine powder, which was then resuspended in 50 mL of 70% (v/v) absolute ethanol and incubated in the dark at 4°C for 72 h. The mixture was then filtered using a glass sintered funnel and the filtrate was dried by evaporation at room temperature using a rota-vapor. The resultant red residue was incubated under vacuum for 4–5 h, following which the mass was recorded. The residue was stored at −20°C and resuspended in 70% (v/v) absolute ethanol prior to use in experiments.

### 2.4 HPLC-MS identification of constituents of *Rhus coriaria* ethanolic extract

RCE was analyzed using the Agilent 6420 Triple Quadrupole LC-MS System (Agilent Technologies, Santa Clara, CA, United States). Before analysis, the sample was filtered through a 0.45 μm syringe filter. The LC-MS system comprised an Agilent EclipsePlus-C18 column (1.8 μm particle size, 2.1 mm × 50 mm length), maintained at 35°C and connected to a tunable UV-Vis detector and the 6420 Triple Quadrupole LC/MS System (all from Agilent Technologies). The mobile phases consisted of 0.1% formic acid (A) and acetonitrile (B), with a gradient profile of 0–2.5 min: 0% B, 2.5–15 min: 20%–100% B, 15–18 min: 100% B, and 18–25 min: 5% B, flowing at 0.2 mL/min. Electrospray ionization source in positive polarity was utilized. LC-MS operational parameters included a capillary voltage of 4 kV, nebulizer pressure set at 45 psi, drying gas flow rate of 11 L/min, and drying temperature maintained at 325°C. Mass detection ranged from 100 to 1,000 Da. This analytical setup facilitated the comprehensive characterization of the constituents of RCE.

**FIGURE 1:**
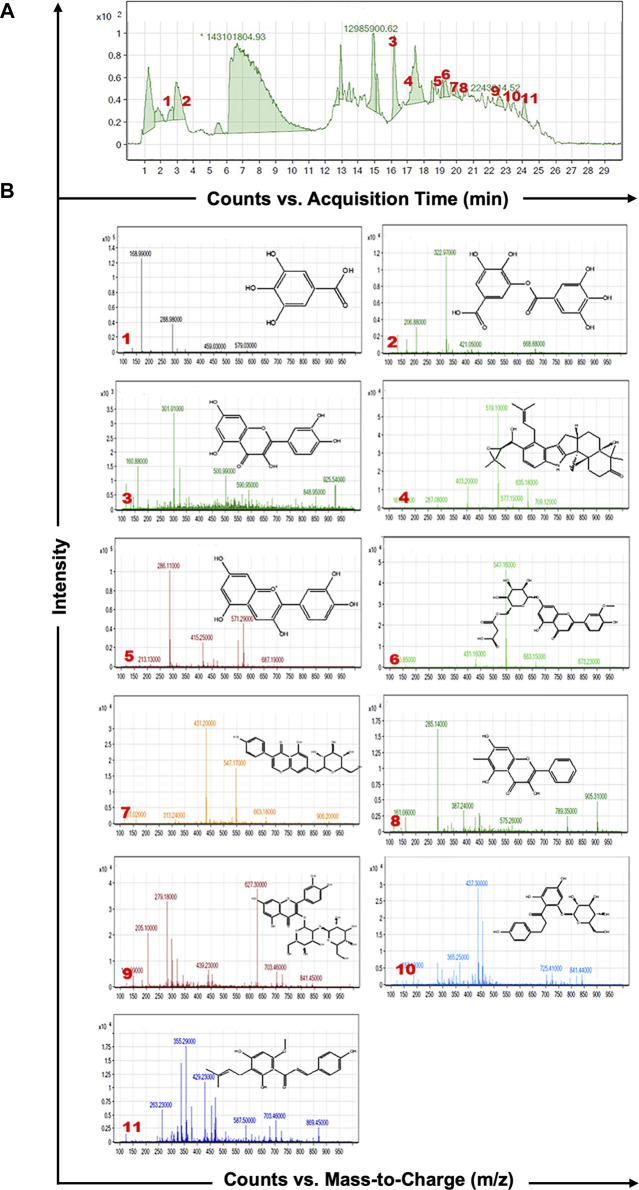
HPLC-MS analysis of *Rhus coriaria* extract. **(A)** The LC-MS chromatographic profile illustrates a comprehensive view of the full scan chromatography of the ethanolic extract of *R coriaria* (prepared as described in *Material and Methods*). **(B)** Extracted ion chromatograms, presenting details of molecular weight, retention time, and chemical structure of the various compounds identified within the sample.

As shown in Figure 1A, HPLC-MS analysis of RCE revealed the presence of several peaks, corresponding to the presence of the following 11 compounds (Figure 1B; Table 1): Gallic Acid (MW: 170.12 g/mol; RT: 2.8 min), Digallic Acid I (MW: 322.22 g/mol; RT: 3.98 min), Quercetin (MW: 302.23 g/mol; RT: 16.08 min), Sespendole (MW: 519.7 g/mol; RT: 17.11 min), Cyanidin (MW: 287.24 g/mol; RT: 19.01 min), Chrysoeriol 7-O-(6″-malonyl-glucoside) (MW: 548.4 g/mol; RT: 19.2 min), Genistin (MW: 432.4 g/mol; RT: 19.59 min), Methylgalangin (MW: 284.26 g/mol; RT: 20.37 min), Quercetin 3-sophoroside (MW: 626.51 g/mol; RT: 22.43 min), Phloretin 2′-glucoside (MW: 436.42 g/mol; RT: 23.01 min), and Xanthohumol (MW: 354.47 g/mol; RT: 23.94 min).

**TABLE 1:** Characterization of the chemical constituents of ethanolic extract of *Rhus coriaria* using HPLC-MS analysis.

S. No	RT (min)	Compound	Molecular formula	Molecular weight (g/mol)	[M + H]+ (m/z)	[M + H]-(m/z)
1	2.80	Gallic Acid	C7H6O5	170.12	—	168.990
2	3.98	Digallic Acid I	C14H10O9	322.22	—	322.970
3	16.08	Quercetin	C15H10O7	302.23	—	301.010
4	17.11	Sespendole	C33H45NO4	519.7	—	518.100
5	19.01	Cyanidin	C15H11O6+	287.24	286.110	—
6	19.20	Chrysoeriol 7-O-(6″-malonyl-glucoside)	C25H24O14	548.40	—	547.160
7	19.59	Genistin	C21H20O10	432.40	—	431.200
8	20.37	Methylgalangin	C16H12O5	284.26	—	285.140
9	22.43	Quercetin 3-sophoroside	C27H30O17	626.51	—	627.300
10	23.01	Phloretin 2′-glucoside	C21H24O10	436.42	437.300	—
11	23.94	Xanthohumol	C21H22O5	354.47	355.290	—

RT, retention time.

### 2.5 Measurement of cell viability

#### 2.5.1 Metabolic activity

Cell viability was assessed using the CellTiter-Glo Luminescent Cell Viability Assay (Promega Corporation, Madison, WI, United States), which assesses ATP levels as a measure of metabolic activity, according to the manufacturer’s instructions. Briefly, pancreatic cancer cells were seeded at a density of 6,000 cells/well in triplicate in 96-well plates (Corning, Corning, NY, United States) and cultured for 24 h prior to treatment. Subsequently, the cells were treated with or without varying concentrations of RCE for different time points. Luminescent signals were measured using GloMax Discover Microplate Reader (Promega). Data are presented as proportional viability by comparing the viability of the treated cells with the control cells, whose viability was assumed to be 100%.

#### 2.5.2 Cell count

Cell viability was also assessed using the Muse Viability Kit (Millipore), which stains viable and dead cells based on differential permeability to two DNA binding dyes. Briefly, Panc-1 and Mia-PaCa-2 were seeded at a density of 5 × 10^5^ cells/well in 12-well plates (Corning) and cultured for 24 h prior to treatment. Cells were counted using the MuseTM Cell Analyzer (Millipore) on the day of treatment to estimate the number of cells at treatment, designated as Day 0. Cells were treated at varying concentrations of RCE for 24 and 48 h, and counted at the end of the experimental time point.

### 2.6 Colony formation assay

Pancreatic cancer cells were seeded at a density of 450 cells/well in 6-well plates (Corning) and cultured for 7 days until visible colonies were formed. Subsequently, formed colonies were treated with varying concentrations of RCE for 5 days. The media (fresh media or media with RCE) was replaced every 3 days. At the end of the treatment period, i.e., post-treatment day 7 (colony day 12), the colonies were washed thrice with 1X phosphate-buffered saline (PBS), fixed for 15 min with 4% formalin, and then stained for 30 min with 0.1% crystal violet. The stained colonies were photographed.

### 2.7 Cell cycle analysis

The effect of RCE treatment on cell cycle distribution was assessed using the Muse™ Cell Cycle Kit (Millipore), according to the manufacturer’s instructions. Briefly, Panc-1 and Mia-PaCa-2 cells were seeded at a density of 2.5 × 10^5^ cells/dish in 60 mm dishes (Corning) and cultured for 24 prior to treatment. Subsequently, cells were treated with varying concentrations of RCE for 48 h, following which the cells were harvested by trypsinization. The resultant cell pellet was resuspended in 500 µL of 1× PBS and fixed with 500 µL of absolute ethanol for at least 3 h prior to staining with the Muse™ Cell Cycle Reagent for 30 min in the dark at room temperature. The stained cells were analyzed using the Muse™ Cell Cycle Kit (Millipore). The percentage of cells in G0/G1, S and G2/M phases was determined using the FlowJo software (Ashland, OR, United States).

### 2.8 Annexin V/PI apoptosis assay

The effect of RCE treatment on apoptosis induction was assessed using the Annexin V & Dead Cell kit (Millipore), according to the manufacturer’s instructions. Briefly, Panc-1 and Mia-PaCa-2 cells were seeded at a density of 4 × 10^4^ cells/well in 12-well dishes (Corning) and cultured for 24 h prior to treatment. Cells were treated with varying concentrations of RCE for 48 h. Subsequently, both adherent and non-adherent cells were harvested and the resultant cell pellet was resuspended in Annexin V and 7-AAD solution and incubated for 20 min in the dark at room temperature. The stained cells were analyzed using the Muse™ Cell Cycle Kit (Millipore) to count the number of viable, early apoptotic, and late apoptotic cells.

### 2.9 Western blot analysis

Panc-1 and Mia-PaCa-2 cells were seeded at a density of 1.8 × 10^6^ cells/dish in 100 mm dishes (Corning) and cultured for 24 h prior to treatment. Cells were then treated with varying concentrations of RCE for 48 h or as indicated, following which the non-adherent cells were pelleted and the adherent cells were washed with ice-cold 1× PBS, scraped, and pelleted by centrifugation. The total cell pellet (adherent and non-adherent cells) was lysed in RIPA lysis buffer (Pierce, Waltham, MA, United States) supplemented with protease/phosphatase inhibitors (Roche, Basel, Switzerland), incubated for 15 min on ice, sonicated, and centrifuged for 30 min at 13,200 rpm at 4°C. The supernatant was collected as the total protein lysate and protein concentration was quantified using a BCA protein assay kit (Thermo Fisher Scientific, Waltham, MA, United States). Proteins (15 µg) were resolved on 6%–15% SDS–PAGE gels alongside PageRuler Plus Prestained Protein Ladder (Thermo Fisher Scientific) and then transferred onto methanol-activated PVDF membranes (Thermo Fisher Scientific). Membranes were blocked with 5% non-fat dry milk in 1× PBS with 0.1% Tween20 (blocking buffer) for 45 min at room temperature. Subsequently, membranes were incubated with the specific primary antibodies in blocking buffer overnight at 4°C, and then with the corresponding HRP-conjugated secondary antibodies in 3% non-fat dry milk in 1× PBS with 0.1% Tween-20 for 30 min at room temperature. Immunoreactive bands were visualized using SuperSignal™ West Femto chemiluminescent substrate (Thermo Fisher Scientific) on the C-DiGit blot scanner (LiCOR, Lincoln, NE, United States) and quantified using ImageJ (National Institutes of Health). Additionally, membranes were stripped when needed using the Restore Western blot stripping buffer (Thermo Fisher Scientific), according to the manufacturer’s instructions.

### 2.10 *In vivo* chick embryo tumor growth assay

The chick embryo tumor growth assay was performed as previously described (Alsamri et al., 2019) with some modifications. Briefly, fertilized eggs were incubated at 37.5°C and 50% humidity. At embryonic day 3 (E3), the chorioallantoic membrane (CAM) was dropped by aspirating 1.5–2 mL of albumin, through a hole opposite to the round wide egg side, and a 1 cm^2^ window was cut in the eggshell above the CAM. At E9, pancreatic cancer cells were detached by trypsinization, washed with complete medium, and resuspended in 70% Matrigel. A 100-µL inoculum of 1 × 10^6^ Panc-1 or Mia-PaCa-2 cells was innoculated onto the CAM of each egg; eggs were randomized in two groups of 17–18 eggs for each cell line. Two days later, tumors that were detectable were treated every second day at E11, E13 and E15 by dropping 100 µL of the vehicle (PBS with 0.01% of ethanol) or RCE (50 mg/kg). At E17, the upper portion of the CAM was removed, washed with PBS, and then the tumors were carefully cut away from normal CAM tissues and weighed to determine the impact of RCE on tumor growth. All data collected were used in statistical analysis.

The *in ovo* tumor xenograft experiments were performed in accordance with the protocol approved by the United Arab Emirates University Animal Ethics Committee (Permit No. ERA_2022_2154 on 14/03/2023).

### 2.11 Statistical analysis

Data are reported as the mean ± SEM and were analyzed using one-way ANOVA with LSD’s Post-Hoc test for multiple comparisons. Tumor volume data were assessed using the unpaired Student’s t-test. A *p*-value of less than 0.05 indicates statistical significance. We conducted all experiments at least three times, with the exception of the *in ovo* assay.

## 3 Results

### 3.1 *Rhus coriaria* extract inhibits the cellular viability of human pancreatic cancer cells

**FIGURE 2:**
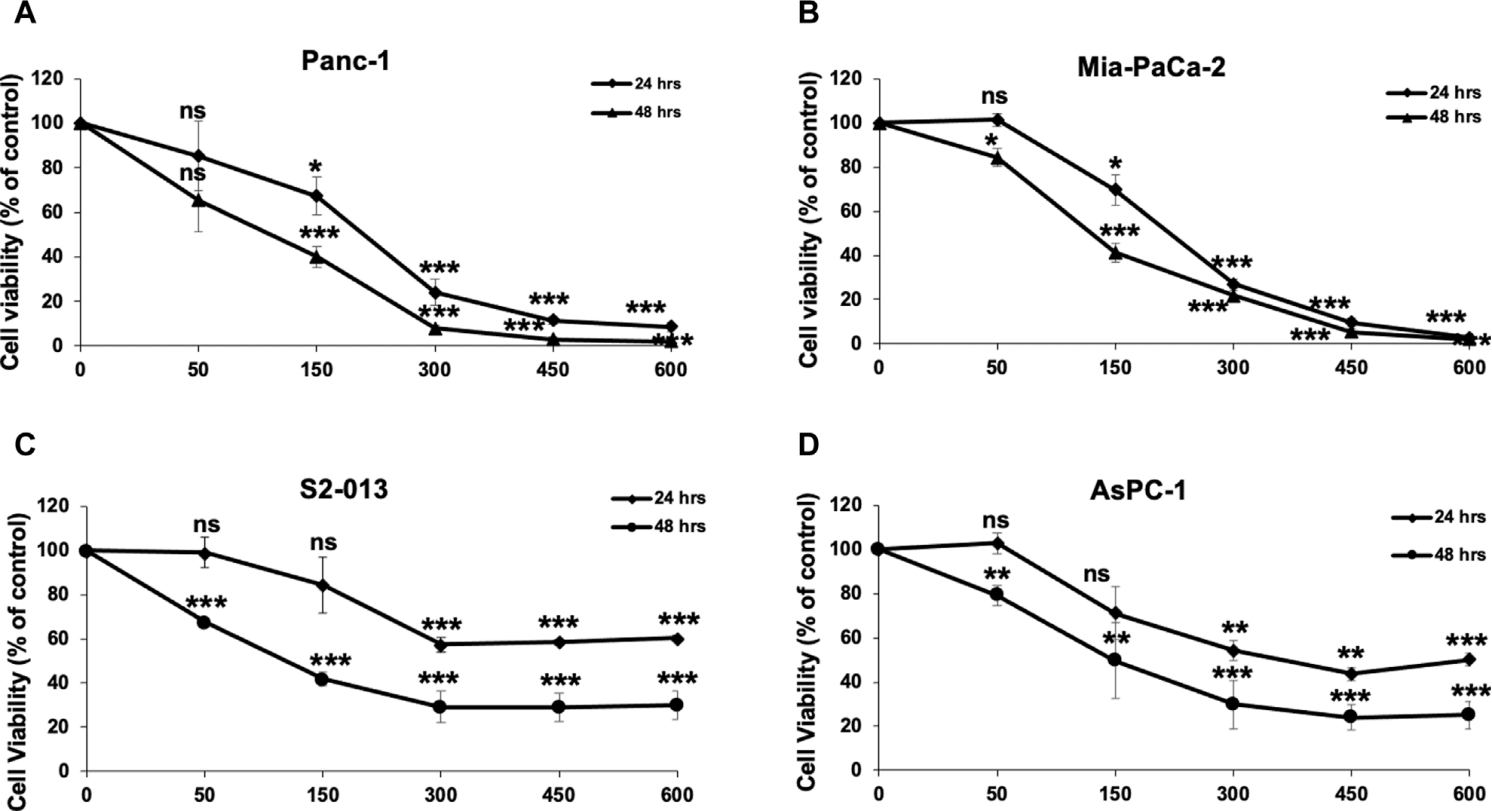
*Rhus coriaria* extract inhibits the cellular viability of human pancreatic cancer cells. **(A)** Panc-1, **(B)** Mia-PaCa-2, **(C)** S2-013, and **(D)** AsPC-1 pancreatic cancer cells were treated with the indicated concentrations of RCE for 24 and 48 h. Cellular viability was measured as described in the *Materials and Methods* section. Data represent the mean of at least three independent experiments carried out in triplicate, and were analyzed using one-way ANOVA followed by LSD Post-Hoc test (*p < 0.05, **p < 0.01, ***p < 0.001).

To examine the anticancer activity of RCE on human pancreatic ductal adenocarcinoma, we measured the effect of varying concentrations of RCE (0, 50, 150, 300, 450, and 600 μg/mL) on the proliferation of four human pancreatic cancer cell lines Panc-1 (Figure 2A), Mia-PaCa-2 (Figure 2B), S2-013 (Figure 2C), and AsPC-1 (Figure 2D), using an assay that assesses cellular metabolic activity. We found RCE treatment significantly decreased the cellular viability of all four pancreatic cancer cell lines in a concentration- and time-dependent manner. Interestingly, Panc-1 and Mia-PaCa-2 were more sensitive to RCE treatment than S2-013 and AsPC-1, which was noticeable starting at 300 μg/mL of RCE at both time points. This can potentially be attributed to genomic differences between these cell lines that affect susceptibility/response to RCE treatment. Collectively, these findings suggest that RCE inhibits the proliferation of pancreatic cancer cells *in vitro*.

### 3.2 *Rhus coriaria* extract inhibits the growth human pancreatic cancer colonies

**FIGURE 3:**
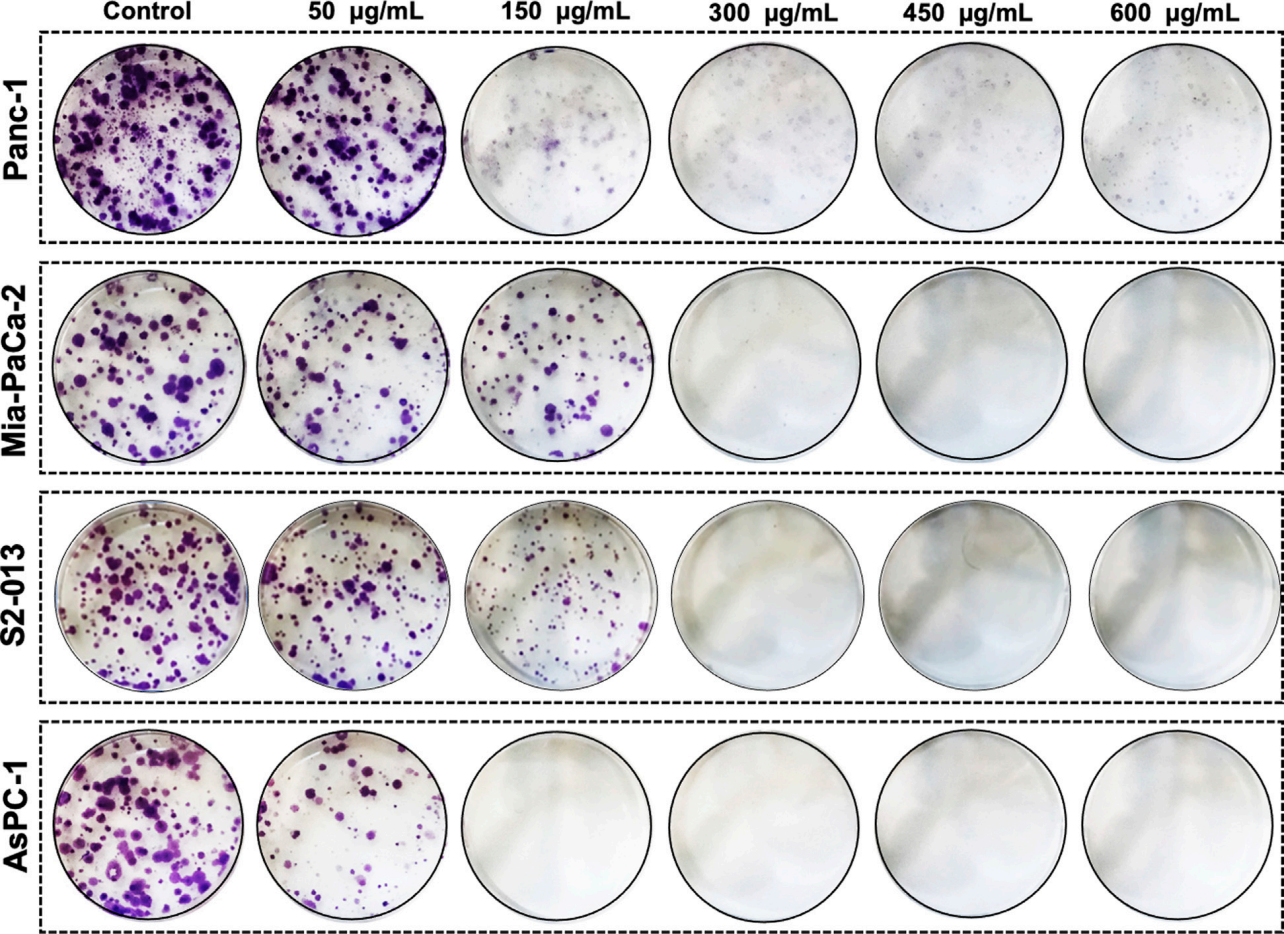
*Rhus coriaria* extract inhibits the colony growth of human pancreatic cancer cells. Panc-1, Mia-PaCa-2, S2-013, and AsPC-1 pancreatic cancer cells were allowed to form colonies in normal media for 7 days as described in the *Materials and Methods* section. Formed colonies were then treated with increasing concentrations of RCE and allowed to grow for seven more days, prior to staining with crystal violet staining.

To further confirm the anticancer potential of *R. coriaria*, we investigated the effect of RCE on the proliferative capacity of formed human pancreatic cancer cell colonies. To this end, Panc-1, Mia-PaCa-2, S2-013, and AsPC-1 cells were seeded at low densities and cultured for 7 days until visible colonies were formed, which were then treated for 7 days with varying concentrations of RCE (0, 50, 150, 300, 450, and 600 μg/mL). As shown in Figure 3, treatment with RCE decreased the number and size of Panc-1, Mia-PaCa-2, S2-013, and AsPC-1 colonies in a concentration-dependent manner. In all four human pancreatic cancer cell lines, the proliferative growth of formed colonies was completely inhibited starting at 300 μg/mL. Moreover, microscopic examination of the treated colonies showed disintegrated colonies even at lower concentrations (data not shown), which is indicative of massive cell death. Collectively, these findings further confirm the antiproliferative/growth inhibitory effect of RCE on human pancreatic cancer cells.

### 3.3 *Rhus coriaria* extract induces G1 arrest in human pancreatic cancer cells

**FIGURE 4:**
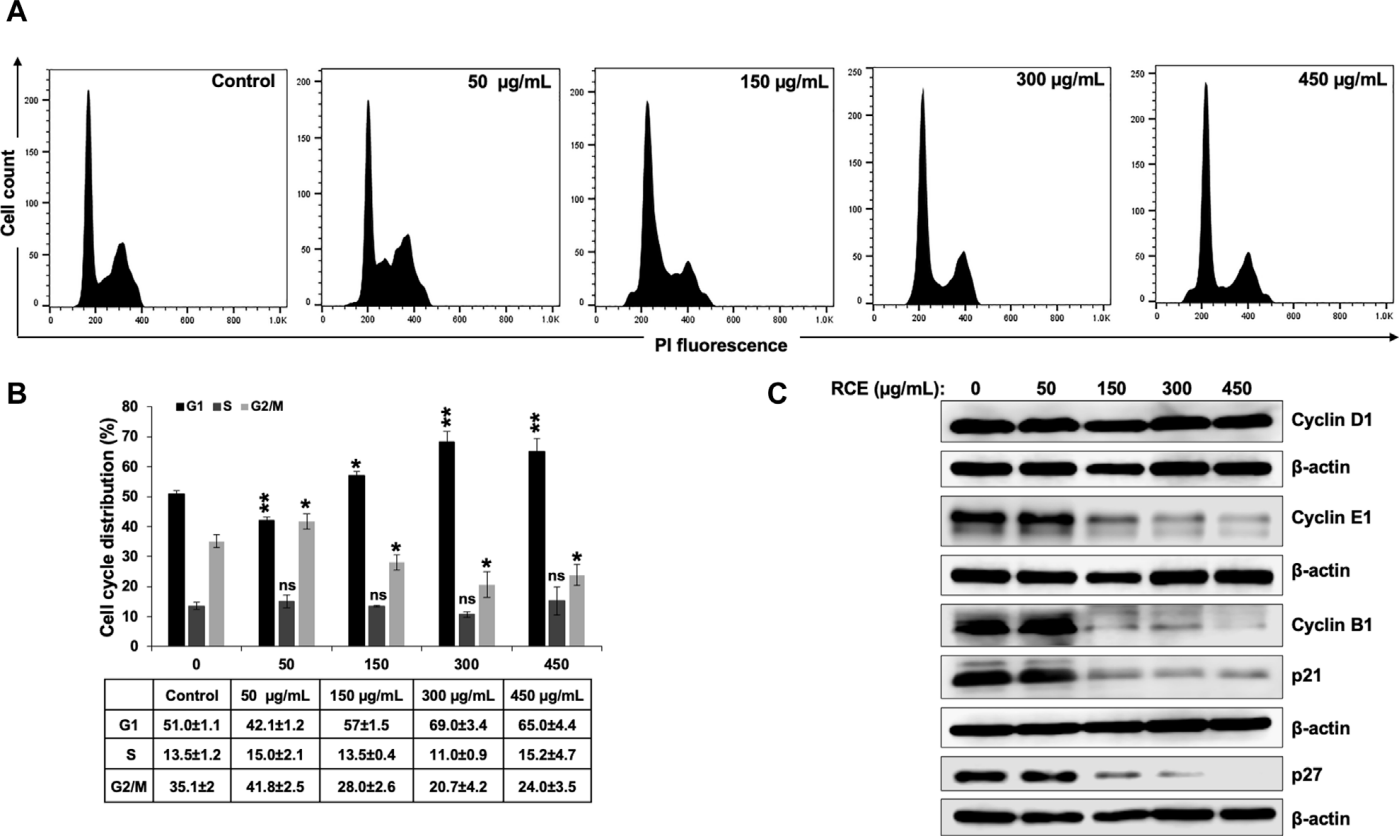
*Rhus coriaria* extract induces G1 cell cycle arrest in Panc-1 pancreatic cancer cells. **(A,B)** Panc-1 cells were treated with RCE at the indicated concentrations for 48 h and cell cycle distribution was analyzed with Muse TM Cell Analyzer as described in the *Materials and Methods* section. Data are representative of three independent experiments performed in triplicate, and were analyzed using one-way ANOVA (*p < 0.05, **p < 0.01). **(C)** Panc-1 cells were treated with the indicated concentrations of RCE for 48 h and the protein levels of Cyclin D1, Cyclin E1, Cyclin B1, p21, and p27 were examined by Western blotting.

To investigate the potential mechanism(s) underlying the observed antiproliferative effect of RCE on human pancreatic cancer cells, we first examined its effect on cell cycle progression. To this end, Panc-1 (Figure 4) and Mia-PaCa-2 (Supplementary Figure S1) were treated with the indicated concentrations of RCE for 48 h and cell cycle distribution was then analyzed. We found that RCE treatment significantly increased the G1 population in Panc-1 (Figures 4A, B) in a concentration-dependent manner; with 51.0 ± 1.1% cells in the G1 population in control cells compared to 57% ± 1.5% and 69.0 ± 3.4% in cells treated with 150 and 300 μg/mL RCE, respectively, for 48 h. Similarly, RCE treatment also increased the G1 population in Mia-PaCa-2 cells (Supplementary Figure S1). These findings are consistent with our previous report as RCE induced G1 arrest in triple negative breast cancer cells (El Hasasna et al., 2015).

To further confirm that RCE impairs cell cycle progression and induces G1 arrest in human pancreatic cancer cells, we analyzed the protein levels of cell cycle-associated proteins. We found that RCE treatment did not affect Cyclin D1 levels in Panc-1 cells (Figure 4C). Cyclin D1 is a key cyclin that regulates entry of cells into G1 phase (Vermeulen et al., 2003; Huber et al., 2021). The lack of effect of RCE treatment on Cyclin D1 levels suggests that RCE does not impair entry into G1 phase, but rather impairs cell cycle progression from G1 to S phase. Consistent with this hypothesis, we found that RCE treatment dose-dependently decreased the protein levels of Cyclin E1 and Cyclin B1 in Panc-1 cells (Figure 4C). Cyclin E1 and Cyclin B1 regulate G1 to S transition and mitosis, respectively, through their respective association with CDK2 and CDK1 (Vermeulen et al., 2003; Huber et al., 2021). Interestingly, we found that RCE treatment decreased the protein levels of CDK inhibitors p21 and p27 after 48 h treatment at concentrations of RCE that induce G1 arrest in Panc-1 cells (Figure 4C). This finding suggests that RCE induces G1 arrest independent of p21 and p27 induction in Panc-1 cells. In accordance with our findings in Panc-1 cells, the same pattern of aberration of cell cycle associated proteins was also noted in RCE-treated Mia-PaCa-2 cells (Supplementary Figure S1). Collectively, these findings confirm that disruption of cell cycle progression from G1 to S phase is a mechanism underlying the antiproliferative effect of RCE in human pancreatic cancer cells.

### 3.4 *Rhus coriaria* extract induces apoptosis in human pancreatic cancer cells

**FIGURE 5:**
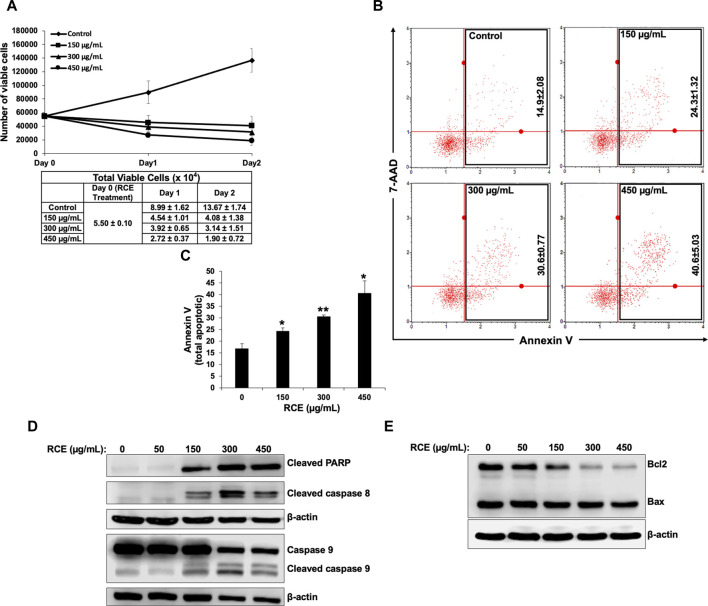
*Rhus coriaria* extract induces apoptosis in Panc-1 pancreatic cancer cells. **(A)** Panc-1 cells were treated with the indicated concentrations of RCE for 24  h and 48 and cell viability was monitored using the Muse cell analyzer as described in the *Materials and Methods* section. Data represent the mean ± SEM of three independent experiments. **(B,C)** Panc-1 cells were treated with the indicated concentrations of RCE for 48 h. Detached and adherent cells were collected, stained with Annexin V and 7-AAD, and the total apoptotic cells were counted with the MuseTM Cell Analyzer as described in the *Materials and Methods* section. Data represent the mean ± SEM of three independent experiments and were analyzed using one-way ANOVA followed by LSD Post-Hoc test (*p < 0.05, **p < 0.01). **(D)** Panc-1 cells were treated with the indicated concentrations of RCE for 48 h and the protein levels of cleaved PARP, cleaved caspase 8, and pro- and cleaved caspase 9, were examined by Western blotting. **(E)** Panc-1 cells were treated with the indicated concentrations of RCE for 48 h and the protein levels of Bax and Bcl2 were examined by Western blotting.

Next, we investigated whether induction of apoptosis contributes to the observed antiproliferative effect of RCE on human pancreatic cancer cells. To this end, we reconfirmed the antiproliferative effect of *R. coriaria* on Panc-1 pancreatic cells using an assay that differentially stains viable and dead cells based on their permeability to two DNA binding dyes. We found that consistent with the results of the metabolic cell viability assay (Figure 2A), RCE treatment decreased the number of viable Panc-1 cells in a concentration- and time-dependent manner, when compared to the number of cells counted at the day of treatment (day 0) (Figure 5A). This finding is suggestive of massive cell death, and hence we used Annexin/7-AAD staining to confirm whether the observed decrease in the number of viable Panc-1 cells was associated with the induction of apoptosis by RCE. We found that RCE treatment significantly increased the total percentage of apoptotic cells in a concentration-dependent manner starting at 150 μg/mL RCE (Figures 5B, C), suggesting that RCE induced apoptosis. We further confirmed induction of apoptosis by RCE by assessing accumulation of cleaved PARP in RCE-treated Panc-1 cells. As shown in Figure 5D, RCE treatment increased the levels of cleaved PARP in a concentration-dependent manner (Figure 5D). Collectively, these findings confirmed that RCE induces apoptosis in human pancreatic cancer cell.

Next, we sought to characterize the mechanism(s) underlying RCE-mediated induction of apoptosis. To this end, we assessed the protein levels of initiator caspases 8 and 9 and executioner caspases 3 and 7 in RCE-treated Panc-1 (Figure 5D) and Mia-PaCa-2 (Supplementary Figure S2). We found that RCE activated both the extrinsic and intrinsic pathways of apoptosis as evidenced by activation of caspase 8 and 9, respectively. Collectively, these findings suggest that *R. coriaria* induces caspase-dependent apoptosis in human pancreatic cancer cells. Given that RCE activated the intrinsic pathway of apoptosis, we assessed the levels of Bcl-2 and Bax, whose ratio represents an apoptotic switch. Bcl-2 is a pro-survival protein that inhibits the activation of the antiapoptotic protein Bax, which mediates mitochondria-dependent apoptosis through mitochondrial outer membrane permeabilization (Adams and Cory, 2007). As shown in Figure 5E, Bcl-2 levels decreased in a concentration-dependent manner in RCE-treated Panc-1 cells, whereas Bax levels were unaffected (Figure 5E). This finding suggests that RCE regulated the Bax/Bcl-2 apoptotic switch to activate the intrinsic pathway of apoptosis in human pancreatic cancer cells.

### 3.5 *Rhus coriaria* extract induces Beclin-1-independent autophagy in human pancreatic cancer cells

**FIGURE 6:**
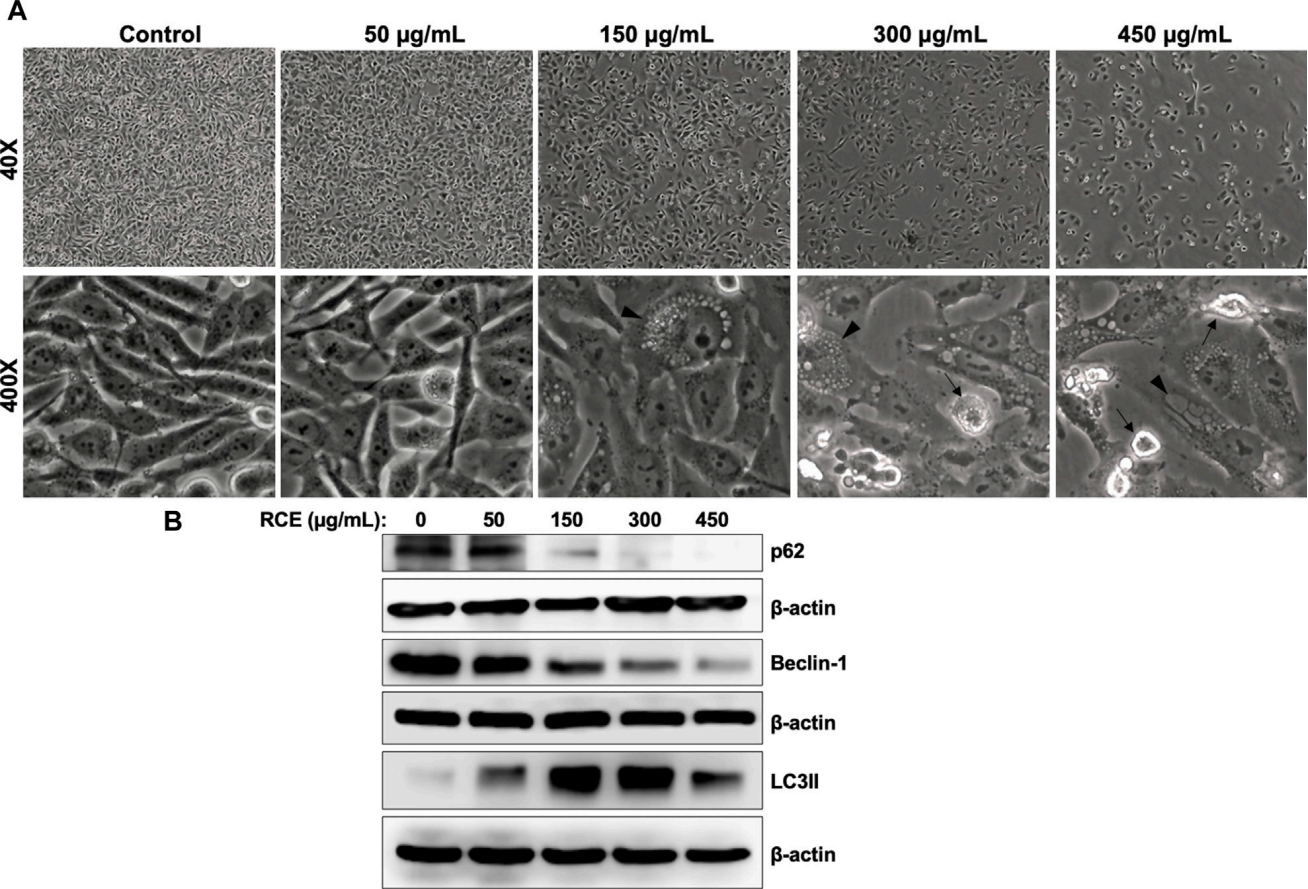
*Rhus coriaria* extract induces Beclin-1-independent autophagy in Panc-1 pancreatic cancer cells. **(A)** Panc-1 cells were treated with the indicated concentrations of RCE for 48 h. Morphological changes were examined under EVOS XL Core Cell Imaging System (Life Technologies). Arrowheads, cytoplasmic vacuolation; arrows, apoptotic cells. **(B)** Panc-1 cells were treated with the indicated concentrations of RCE for 48 h and the protein levels of p62, LC3-II, and Beclin-1 were examined by Western blotting.

On microscopic examination of RCE-treated Panc-1 cells (Figure 6A), we noted cytoplasmic vacuolation (arrow heads), which is potentially suggestive of autophagy. Moreover, we previously reported that RCE induces autophagy in triple negative breast cancer (El Hasasna et al., 2015) and colon cancer cells (Athamneh et al., 2017). Hence, to confirm the autophagic nature of the observed vacuoles in RCE-treated human pancreatic cancer cells, we examined the protein levels of autophagy markers p62 (Sequestosome 1) and LC3-II. The lipidation of LC3-I to LC3-II is a characteristic marker of autophagy induction, whereas the degradation of the ubiquitin-binding protein p62 is a characteristic marker of autophagic flux (Klionsky et al., 2021). We found that RCE treatment induced a concentration-dependent increase in LC3-II levels starting at 150 μg/mL in Panc-1 (Figure 6B) and Mia-Paca-2 (Supplementary Figure S3) cells and a concentration-dependent decrease in p62 levels starting at 150 μg/mL in Panc-1 cells (Figure 6B). These findings confirm that RCE induces autophagic flux in human pancreatic cancer cells. We also assessed the levels of Beclin-1, a component of the PI3KC3 autophagic complex (Klionsky et al., 2021), as previously we reported contrasting results: canonical Beclin-1-dependent autophagy in triple negative breast cancer (El Hasasna et al., 2015) and non-canonical Beclin-1-independent autophagy colon cancer cells (Athamneh et al., 2017). Consistent with our previous findings in colon cancer cells (Athamneh et al., 2017), RCE treatment decreased Beclin-1 levels in a concentration-dependent manner in Panc-1 (Figure 6B) and Mia-Paca-2 (Supplementary Figure S3) cells. Collectively, these findings suggest that RCE induces Beclin-1-independent autophagy in human pancreatic cancer cells.

### 3.6 *Rhus coriaria* inhibits the AKT/mTOR/p70S6K pathway in human pancreatic cancer cells

**FIGURE 7:**
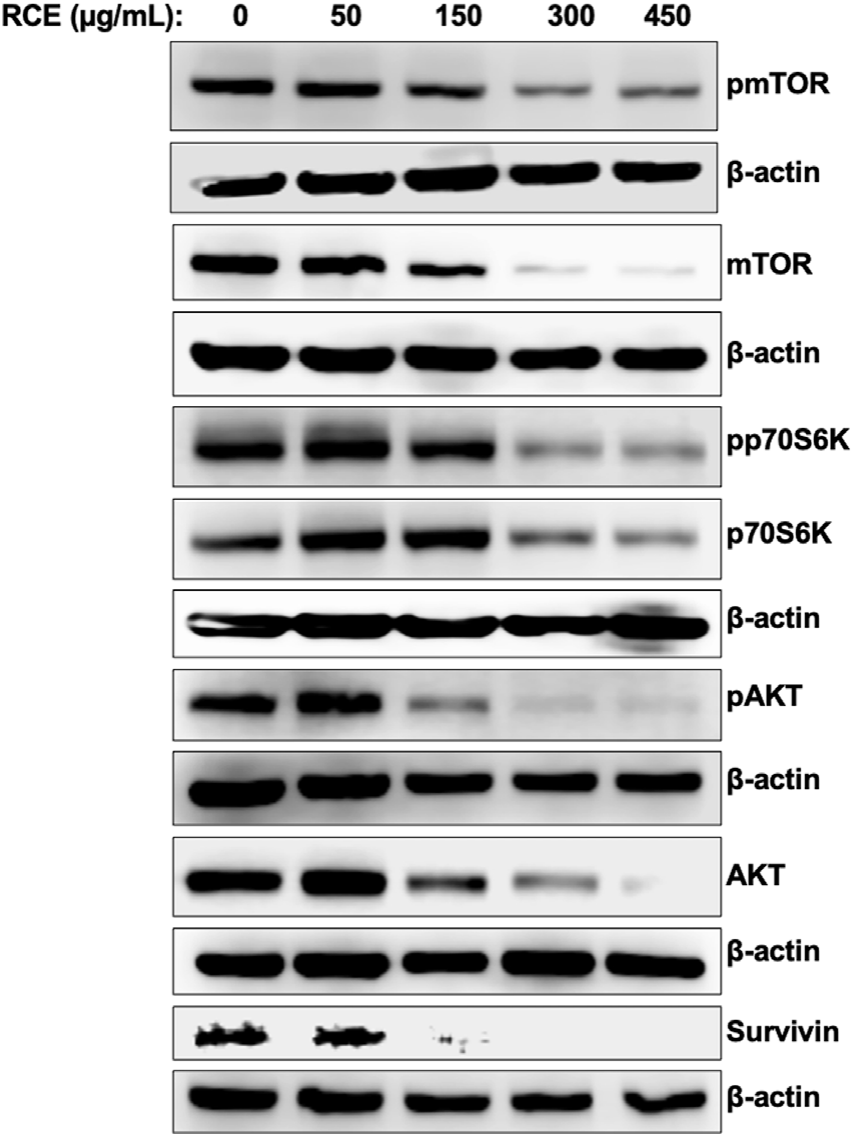
*Rhus coriaria* extract inhibits the AKT/mTOR/p70S6K pathway in Panc-1 pancreatic cancer cells. Panc-1 cells were treated with the indicated concentrations of RCE for 48 h. The protein levels of phosphorylated (p-) and total mTORC1, p- and total AKT, p- and total p70S6K, and survivin were examined by Western blotting.

Next, we sought to characterize the mechanism(s) through which *R. coriaria* induces autophagy in human pancreatic cancer cells. mTORC1 is the main negative regulator of autophagy in mammalian cells, and its activation is regulated by the upstream regulator AKT (Paquette et al., 2018). Hence, we examined the effect of RCE treatment on the protein levels of total and phosphorylated mTORC1 and AKT. As shown in Figure 7, RCE treatment led to a concentration-dependent decrease in the protein levels of both total and phosphorylated (Ser2448) mTORC1, suggesting inhibition of mTORC1 activity in RCE-treated Panc-1 (Figure 7) and Mia-PaCa-2 (Supplementary Figure S3) cells. Consistently, total and phosphorylated AKT protein levels were also decreased in a concentration-dependent in RCE-treated Panc-1 (Figure 7) and Mia-PaCa-2 (Supplementary Figure S3) cells. These findings were consistent with our previous reports in colon cancer cells (Athamneh et al., 2017).

Phosphorylation of p70S6K at Thr389 is a hallmark of mTORC1 activation, and moreover, this kinase also plays a role in G1 cell cycle progression (Pópulo et al., 2012). We found that RCE treatment inhibited the phosphorylation of p70S6K at Thr389 and decreased total p70S6K protein levels in a concentration-dependent manner in RCE-treated Panc-1 (Figure 7) and Mia-PaCa-2 (Supplementary Figure S3) cells. Interestingly, we also found that the protein levels of survivin, an anti-apoptotic protein whose mRNA translation is regulated by mTORC1/p70S6K activation (Vaira et al., 2007), was decreased in RCE-treated Panc-1 cells (Figure 7). Collectively, these findings further confirm that RCE inhibits the AKT/mTOR pathway in human pancreatic cancer cells to induce autophagy. Furthermore, downstream of this, inhibition of p70S6K activation and downregulation of survivin can be implicated in RCE-induced G1 cell cycle arrest (Figure 4; Supplementary Figure S1) and apoptosis (Figure 5; Supplementary Figure S2) in human pancreatic cancer cells.

### 3.7 *Rhus coriaria*-mediated induction of autophagy precedes apoptosis in human pancreatic cancer cells

**FIGURE 8:**
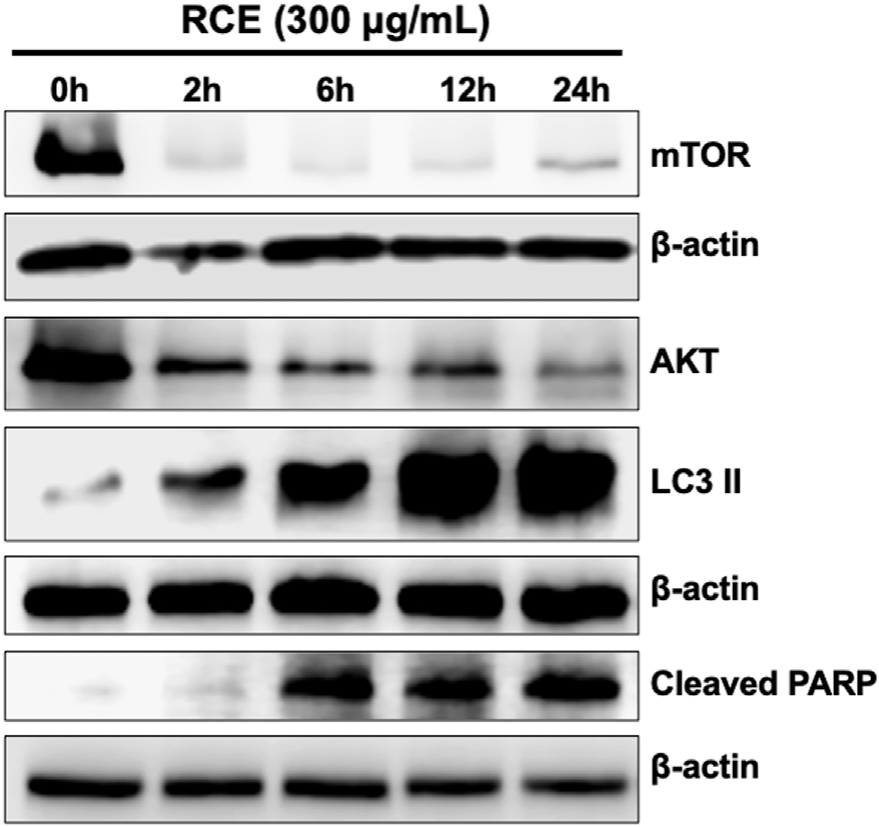
*Rhus coriaria* extract-induced autophagy precedes in Panc-1 pancreatic cancer cells. Panc-1 cells were treated with 300 μg/mL RCE at the indicated time-points (2, 6, 12, and 24 h). The protein levels of mTOR, AKT, LC3II and cleaved PARP were examined by Western blotting.

*Rhus coriaria* induced both autophagy and apoptosis, which represent programmed cell death type I and II, respectively, in human pancreatic cancer cells. Previously, we reported that autophagy is an early event that precedes apoptosis in RCE-treated colon cancer cells (Athamneh et al., 2017). Hence, herein, we sought to clarify the timeline of induction of autophagy and apoptosis in RCE-treated human pancreatic cancer cells. To this end, we performed a time-course experiment (0, 2, 6, 12, and 24 h) with 300 μg/mL RCE and assessed the protein levels of autophagy (LC3II, mTOR, and AKT) and apoptosis (cleaved PARP) markers. As shown in Figure 8, 300 μg/mL RCE induced autophagy as early as 2 h after treatment which was evidenced by increased LC3II levels level in Panc-1 cells. This was concomitant with decreased levels of mTORC1 and AKT 2 h post-treatment with 300 μg/mL RCE, further confirming that the inhibition of the AKT/mTOR pathway is involved in RCE-mediated autophagy. On the other hand, PARP cleavage was noted starting at 6 h post-treatment with 300 μg/mL RCE in Panc-1 cells (Figure 8). Similar findings were also noted in 300 μg/mL RCE-treated Mia-PaCa-2 cells with LC3II and cleaved PARP accumulation at 2 h and 6 h post-treatment, respectively (Supplementary Figure S4). Collectively, these findings confirm that autophagy precedes apoptosis in the mechanisms underlying the antiproliferative effects of RCE on human pancreatic cancer cells.

### 3.8 *Rhus coriaria* inhibits the growth of human pancreatic cancer in a chick embryo xenograft model

**FIGURE 9:**
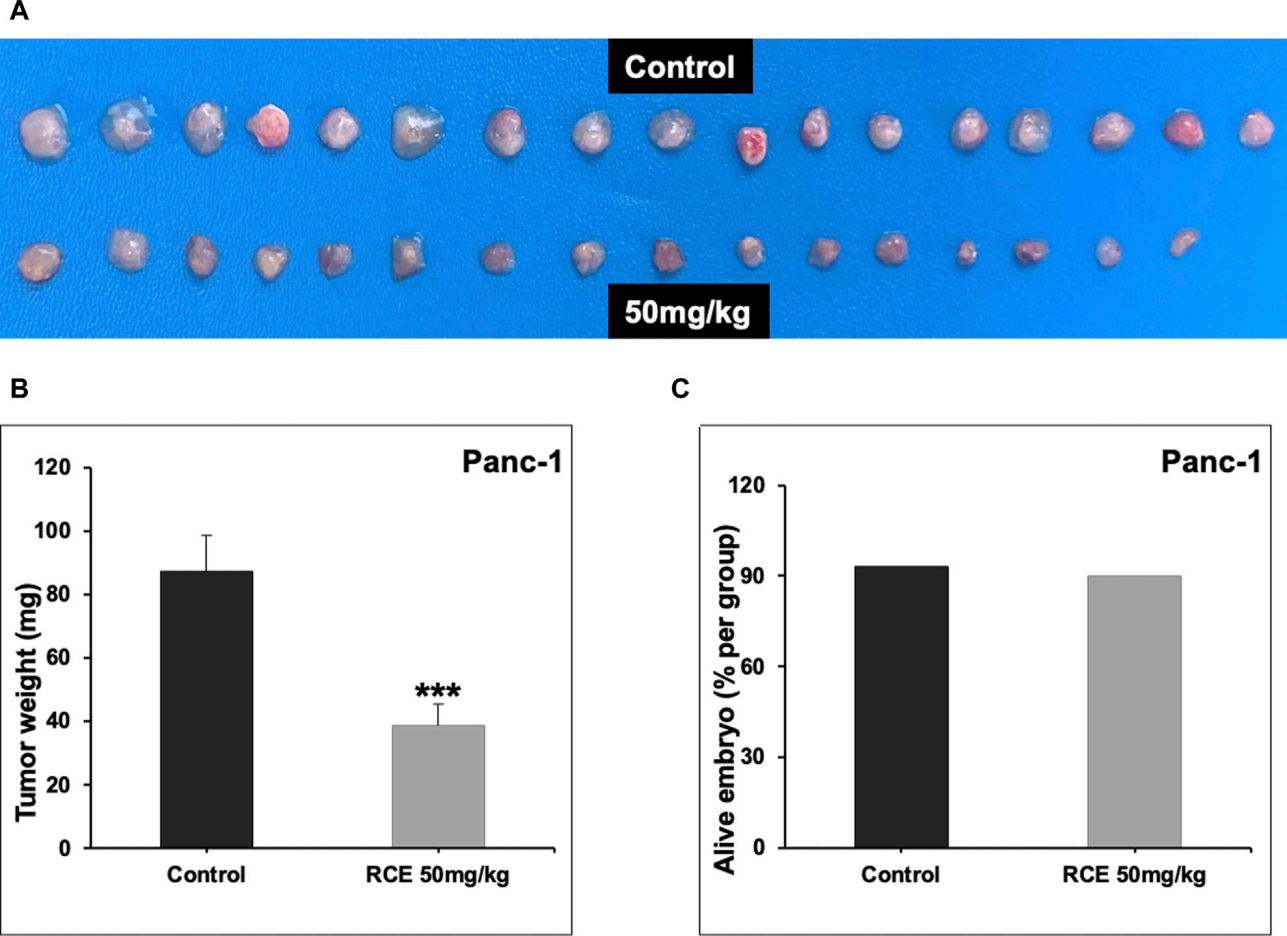
*Rhus coriaria* extract inhibits the growth of Panc-1 pancreatic cancer cells in a chick embryo xenograft model. **(A)** Panc-1 cells were inoculated on the chorioallantoic membrane of 9 days (E9) chick embryos. Tumors were treated every 48 h with 50 mg/kg of RCE as described in the *Materials and Methods* section. At E17, tumors were collected and weighted. **(B)** Tumor weight (mg) in control- and RCE-treated chick embryos were quantified. **(C)** The number of surviving control- and RCE-treated chick embryos were quantified. Data represent the mean ± SEM (***p < 0.001).

Next, we confirmed the pharmacological relevance of our *in vitro* data using a chick embryo xenograft model. To this end, we inoculated Panc-1 (Figure 9A) and Mia-PaCa-2 (Supplementary Figure S5) pancreatic cancer cells on the CAM of fertilized chicken eggs and then treated the formed tumors with the control (PBS with 0.01% of ethanol) or 50 mg/kg of RCE. We found that 50 mg/kg of RCE significantly inhibited tumor growth by 64% and 75% for Panc-1 (Figure 9B) and Mia-PaCa-2 (Supplementary Figure S5) cells, respectively, compared with their controls. Noteworthily, there was no difference in the number of surviving control and RCE-treated embryos (Figure 9C), confirming that RCE does not exhibit cytotoxicity *in vivo*. Collectively, these findings confirm the anticancer activity of RCE in human pancreatic cancer cells *in vivo*.

## 4 Discussion

In the present study, we investigated the anticancer effect of *R. coriaria* in a battery of four human pancreatic cancer cell lines. To the best of our knowledge, this is the first report to establish that RCE exerts antiproliferative effects against pancreatic cancer cells, which we also confirmed in a chick embryo xenograft model. RCE’s antiproliferative effects are mediated by different mechanisms including G1 cell cycle arrest, Beclin-1-independent autophagy, and caspase-dependent apoptosis. RCE-induced G1 cell cycle arrest was characterized by downregulation of Cyclin E1, Cyclin B1, p21, and p27. Our results show that, mechanistically, RCE induced Beclin-1-independent autophagy, characterized by downregulation of Beclin-1, through inhibition of the AKT/mTOR pathway. Further downstream of this pathway, RCE inactivated p70S6K, which might be implicated in G1 cell cycle progression, and downregulated survivin, an antiapoptotic protein. In addition, we report here that RCE-induced autophagy preceded apoptosis, which involved both the intrinsic and extrinsic pathways of apoptosis as well as downregulation of Bcl-2.

Programmed cell death, or apoptosis, is a fundamental process that plays critical roles in health and disease. Cells execute apoptotic programs as a defensive mechanism as well as a tightly regulated event to deter aberrant cell growth, which may precipitate various diseases including cancer. Apoptosis can occur via the intrinsic or extrinsic pathways. While the extrinsic pathway involves external stimuli acting through death receptors, such as Fas, the intrinsic one largely depends on intracellular events, such as DNA damage or oxidative stress especially with mitochondrial involvement. In this report, we showed that RCE caused a marked increase in the activity of both pathways in pancreatic cancer cells. Despite the novelty of this findings, it is not surprising given that *R. coriaria* is bountiful in various bioactive molecules known to modulate apoptosis. For instance, quercetin, present in RCE, induces apoptosis in a battery of cancer cell lines, including pancreatic cancer cell lines (Kim et al., 2016). Similarly, other bioactive molecules present in RCE, such as genistein (Xia et al., 2012), xanthohumol (Cykowiak et al., 2021; Palanisamy et al., 2024), and cyanidin (Farhan et al., 2022) are known to induce the same.

One of the key players implicated in regulating apoptotic programs is survivin. With a 16.5 kDa molecular weight, survivin is the smallest member of the inhibitor of apoptosis protein (IAP) family. It plays various roles in normal cell physiology as well as in cancer cell growth and metastasis. It inhibits apoptosis by virtue of its ability to bind to effector and executioner caspases, namely, caspase-3, -7 and -9, and inhibits caspase activity and cell death in cells exposed to diverse apoptotic stimuli (Tamm et al., 1998). Here, we showed that RCE significantly diminished the expression of survivin, thereby contributing to induction of apoptosis. It is important to note here that increased serum levels of survivin have been linked to increased cancer risk (Liping et al., 2010), rendering this protein as a potential biomarker in cancer (Yin et al., 2006; Xie et al., 2016; Martínez-Sifuentes et al., 2022). Our results also show a concomitant decrease in Akt along with the decrease in survivin expression. This is not surprising given that survivin has been shown to be induced by the PI3K/Akt pathway (Li et al., 2023; Wei et al., 2023). Our results are also consistent with reports showing that suppression of the Akt pathway leads to decreased expression of survivin (Cao et al., 2023). Hence, the RCE-induced decrease in survivin expression further cements our earlier reports that *R. coriaria* possesses various anticancer activities and could be an important source of anticancer compounds.

Autophagy is a highly conserved mechanism that cells employ to maintain intracellular homeostasis. Accumulating evidence links autophagy to cancer progression and metastasis (Biswas et al., 2024; Strippoli et al., 2024). Importantly, progression of pancreatic cancer has been associated with dysregulation of autophagy. However, it is critical to note here that autophagy may be a pro-survival or a pro-death signal in pancreatic cancer. For instance, it has been shown that elevated basal levels of autophagy are vital players in pancreatic cancer tumorigenesis (Yang et al., 2011). The genetic and pharmacologic inhibition of autophagy suppressed the growth of pancreatic cancer cell lines *in vitro*, probably as result of ROS production, DNA damage and decreased mitochondrial function (Yang et al., 2011). We and others have previously showed that autophagy is a pro-death signal in other types of cancer, such as breast and colon cancers (El Hasasna et al., 2015; Athamneh et al., 2017; Benhalilou et al., 2019). Herein, our results support a pro-death impact of autophagy. This is in line with reports showing that chemical (using the small molecule YM155) or genetic inhibition of survivin (via siRNA) evokes autophagy-dependent apoptosis in cancer cells (Wang et al., 2011).

Cell cycle progression is orchestrated by various proteins, including cell cycle check point inhibitors, such as p21 and p27 (Coqueret, 2003). These two proteins are known to suppress/halt cell cycle progression, and hence, their increased expression is associated with reduced mitotic activity. However, increasing evidence suggests that cell cycle can be blocked even with no alteration or downregulation of the levels of these two proteins (Cheng et al., 2001; Gartel and Radhakrishnan, 2005). This is in line with our findings for RCE in pancreatic cancer cells. It is important to note that this does not seem to be a universal effect of RCE and may rather be cell context dependent. For instance, we previously showed the RCE inhibits cell cycle in breast cancer cells, with upregulated p21 levels and downregulated p27 cells (El Hasasna et al., 2015). These results in breast cancer cells are in marked contrast to what we report here in pancreatic cancer cells, specifically for p21 levels. Therefore, the effects of an anticancer agent on cell cycle, be it a pure compound or an extract, need to be carefully analyzed; generalizing or extrapolating from one cell type to another may not be warranted. Cyclin E, which regulates the G1/S phase transition, has been reported to be highly expressed in pancreatic cancer and promotes pancreatic adenocarcinoma (Liu Z. et al., 2020), and its downregulation suppresses pancreatic tumorigenesis (Franco et al., 2014; Liu B. et al., 2020). In addition, cyclin E overexpression is considered a predictor of poor outcomes in patients with pancreatic cancer. In addition, dysregulated cyclin E1 expression is associated with chemotherapy resistance (Pang et al., 2020). Similarly, overexpression of cyclin B1, a cell cycle regulator involved in the regulation of G2/M phase, has been consistently correlated with increased pancreatic cancer incidence, much so that it is now considered a prognostic factor for the same (Zhou et al., 2014). Moreover, patients with pancreatic cancer with high cyclin B expression have lower survival rate than those with low cyclin B expression (Zhou et al., 2014). Silencing of cyclin B1 in pancreatic cancer cells was associated the inhibition of cellular proliferation and an increase in the G0/G1 cell population (Zhang et al., 2019). Herein, we report that *R. coriaria* extract downregulated the levels of both cyclin E and cyclin B1 in pancreatic cancer cells. This finding is in agreement with other studies which reported that concomitant decrease in cyclin B1 and cyclin E1 levles is associated with suppression of proliferation of pancreatic cancer cells (Balasubramanian et al., 2005). Taken together, these studies highlight the importance of these cell cycle regulatory molecules, and highlight their potential as tractable targets in the fight against pancreatic cancer.

Autophagy and apoptosis are cellular processes that are intricately linked. Several proteins, including DAPK, JNK, p62, Beclin-1, Bcl-2, among others, are known to play dual roles in autophagy and apoptosis (Gump and Thorburn, 2011; Xi et al., 2022). Notably, Beclin-1, a critical regulator of autophagy interacts with the anti-apoptotic Bcl-2, representing an important cross talk between the two pathways (Gump and Thorburn, 2011; Xi et al., 2022). It is important to note that in the present study, RCE induced Beclin-1-independent autophagy and decreased BCl-2 protein levels in pancreatic cancer cells. This is particularly interestingly, and further supports the argument that RCE-induced autophagy preceded apoptosis in pancreatic cancer cells, as Beclin-1-independent autophagy is reported to play a pro-death role over a pro-survival role through induction of apoptosis (Grishchuk et al., 2011; Sun et al., 2015). Understanding the molecular events involved in the precedence of autophagy over apoptosis by RCE might help tailor RCE-based therapies for pancreatic cancer, given the role of autophagy in pancreatic cancer progression. Autophagy is known to be upregulated in pancreatic ductal adenocarcinoma in particular, and hence, modulating autophagy is an up-and-coming therapeutic approach. However, most studies have focused on the inhibition of autophagy as opposed to further augmentation of autophagy to induce apoptosis (Piffoux et al., 2021). In this aspect, RCE-based therapies represent a promising avenue.

In conclusion, this is the first report demonstrating the anticancer effect of *R. coriaria*, a Mediterranean plant, on the growth of pancreatic cancer cells *in vitro* and *in vivo* (chick embryo xenograft model). These findings add to the existing body of literature that this plant exerts anticancer effects in breast and colon cancers, and further support the assertion that *R. coriaria* may prove to be an important and bountiful resource for various anticancer agents. Equally important, these results in various cancers may support the consumption of this plant and further encourage the public to incorporate it into their regular diet as a preventive approach to reduce the risk and burden of cancer.

## Data Availability

The original contributions presented in the study are included in the article/Supplementary Material, further inquiries can be directed to the corresponding author.
