# Supplementary material for: Rhus coriaria induces autophagic and apoptotic cell death in pancreatic cancer cells
Source: Front Pharmacol. 2024 Jul 30;15:1412565. doi: 10.3389/fphar.2024.1412565 (PMC11319293; doi:10.3389/fphar.2024.1412565)
Supplement: Supplementary file 1 [file DataSheet1.PDF]

Supplementary Material

1 Supplementary Figures

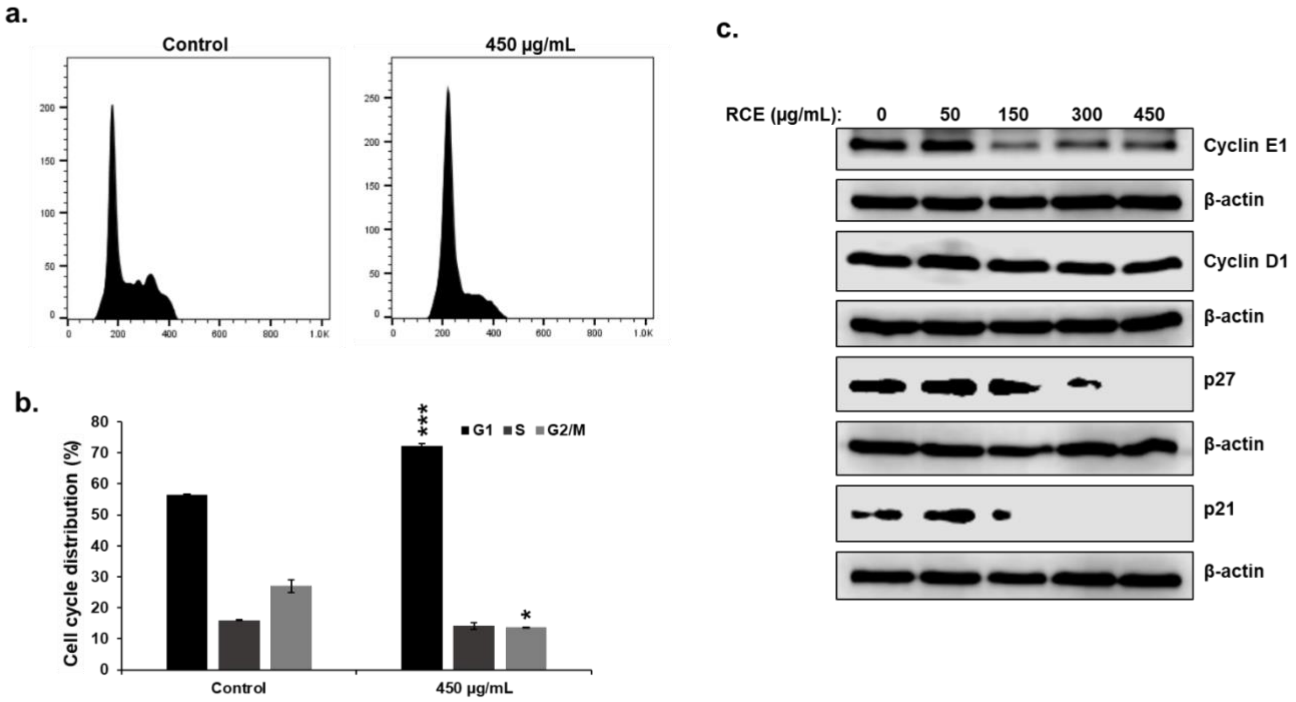

Supplementary Figure 1. *Rhus coriaria* induces G1 cell cycle arrest in Mia-PaCa-2 pancreatic cancer cells. (**A, B**) Mia-PaCa-2 cells were treated with RCE at the indicated concentrations for 48 h and cell cycle distribution was analyzed with Muse TM Cell Analyzer as described in the Materials and Methods section. Data are representative of three independent experiments performed in triplicate. Statistical analysis was performed using one-way ANOVA (*$p < 0.05$, ***$p < 0.001$). (**C**) Mia-PaCa-2 were treated with the indicated concentrations of RCE for 48 h and the protein levels of Cyclin E1, Cyclin D1, p21 and p27 were examined by western blotting.

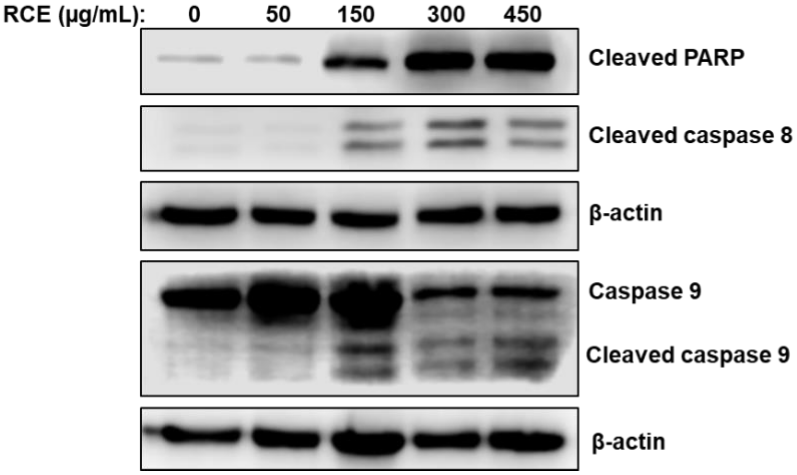

Supplementary Figure 2. *Rhus coriaria* extract induces apoptosis in Mia-PaCa-2 pancreatic cancer cells. Mia-PaCa-2 cells were treated with the indicated concentrations of RCE for 48 h and the protein levels of cleaved PARP, cleaved caspase 8, and pro- and cleaved caspase 9, were examined by western blotting.

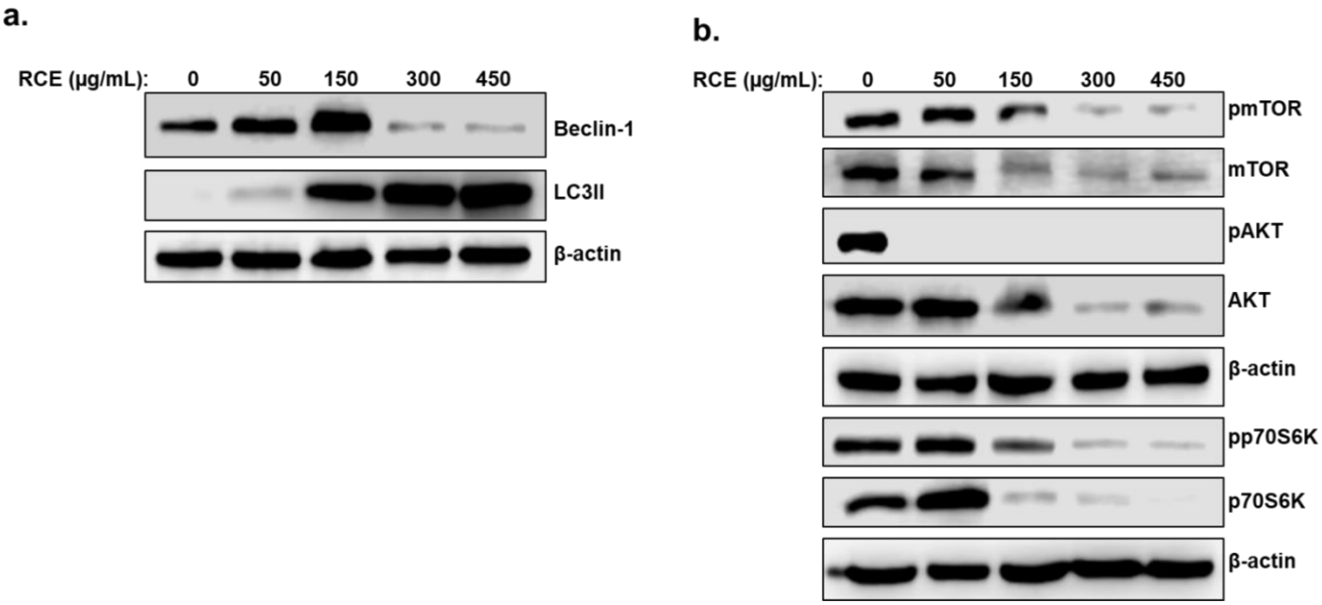

Supplementary Figure 3. *Rhus coriaria* extract induces Beclin-1-independent autophagy in Mia-PaCa-2 cells through inhibition of the AKT/mTOR/p70S6K pathway. Mia-PaCa-2 cells were treated with the indicated concentrations of RCE for 48 h and the protein levels of (**A**) LC3-II, Beclin 2, and phosphorylated (p-) and (**B**) total forms of mTOR, AKT, and p70S6K were examined by western blotting.

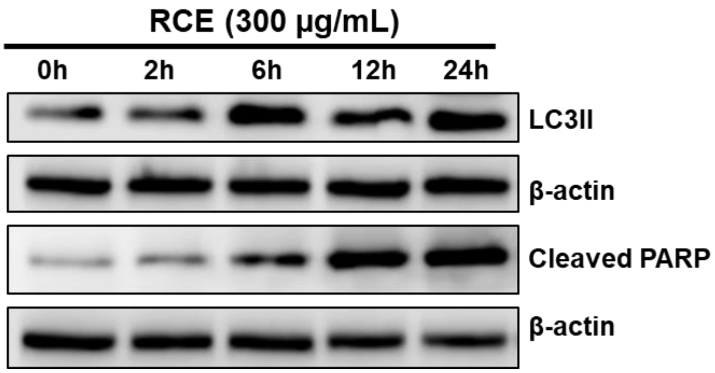

Supplementary Figure 4. *Rhus coriaria*-induced autophagy precedes apoptosis in Mia-PaCa-2 pancreatic cancer cells. Mia-PaCa-2 were treated with 300 µg/mL RCE at the indicated time-points (2, 6, 12, and 24 h). The protein levels of LC3II and cleaved PARP were examined by western blotting.

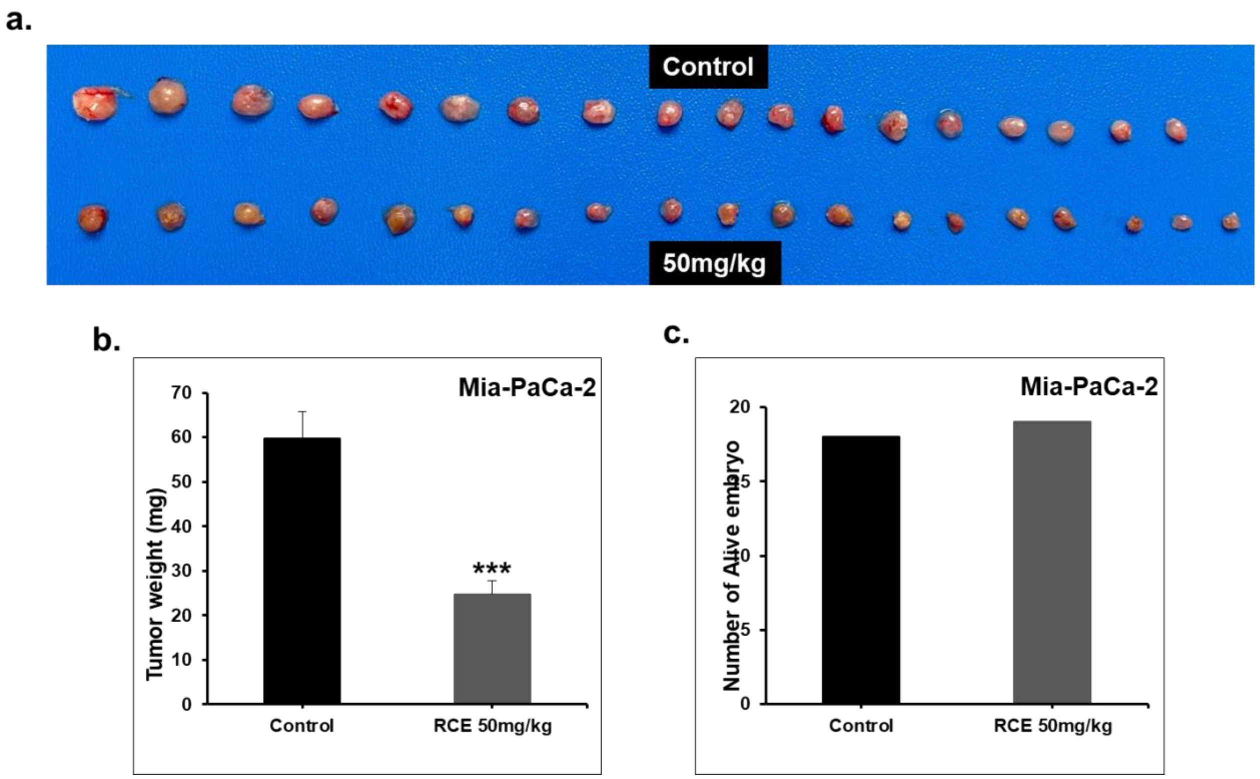

Supplementary Figure 5. *Rhus coriaria* inhibits the growth of Mia-PaCa-2 pancreatic cancer cells in a chick embryo xenograft model. (**A**) Mia-PaCa-2 cells were inoculated on the chorioallantoic membrane of 10 day (E9) chick embryos. Tumors were treated every 48 h with 50 mg/kg of RCE as described in the Materials and Methods section. At E17, tumors were collected and weighted. (**B**) Tumor weight (mg) in control- and RCE-treated chick embryos were quantified. (**C**) The number of surviving control- and RCE-treated chick embryos were quantified. Data represent the mean ± SEM (***$p < 0.001$).
